# Bilateral congenital glaucoma in a child with Nicolaides-Baraitser syndrome: a case report

**DOI:** 10.1097/MS9.0000000000004499

**Published:** 2025-12-04

**Authors:** Yahya A. Alyahya, Ehab Y. Alsirhy, Saeed F. Alwadani, Tariq A. Al-Anazi, Altaf A. Kondkar, Taif A. Azad

**Affiliations:** a Ophthalmology Department, King Saud University, College of Medicine, Riyadh, Saudi Arabia; b Glaucoma and Cataract Consultant, Ophthalmology Department, King Saud University, College of Medicine, Riyadh, Saudi Arabia; c Ophthalmology Consultant, Ophthalmology Department, King Saud University, College of Medicine, Riyadh, Saudi Arabia; d Department of Ophthalmology, Glaucoma Research Chair in Ophthalmology, College of Medicine, King Saud University, Riyadh, Saudi Arabia

**Keywords:** case report, childhood glaucoma, NCBRS, ophthalmology, SMARCA2

## Abstract

**Introduction and importance:**

Nicolaides-Baraitser syndrome (NCBRS) is a rare autosomal dominant disease characterized by developmental delay, distinctive craniofacial features, sparse hair, and is caused by *de novo* mutations in the *SMARCA2* gene.

**Case presentation:**

We report the case of an 11-year-old male with NCBRS presenting with bilateral congenital glaucoma. The diagnosis was based on clinical presentation, such as attention deficit hyperactivity disorder, cognitive delay, and characteristic craniofacial features, and confirmed by the presence of a *de novo* mutation in the SMARCA2 gene through whole-exome sequencing.

**Clinical discussion:**

This case of a child with bilateral congenital glaucoma contributes further evidence of abnormal ocular features associated with the phenotypic spectrum of NCBRS, marking only the third such case reported in the literature.

**Conclusion:**

This rare association of NCBRS with bilateral congenital glaucoma highlights the importance of ophthalmologic screening in patients with NCBRS.

## Introduction

Nicolaides–Baraitser syndrome (NCBRS, OMIM # 601358) is a rare genetic disorder with only 100 cases reported globally^[1,2]^. It is characterized by moderate-to-severe intellectual disability and developmental delays, which may include speech and cognitive developmental impairments[2]. Common phenotypic facial manifestations of affected individuals include a prominent forehead, wide spacing of the eyes or hypertelorism, a short upturned nose with a broad nasal bridge, a long philtrum, and a thin upper lip. Neurological abnormalities include seizures, hypotonia, microcephaly, and structural brain anomalies^[1,3]^. Other associated features are musculoskeletal malformations such as short stature, joint contractures, and spinal scoliosis[3]. Other appearances include feeding issues, gastrointestinal difficulties, hearing loss, and vision disturbances[4]. Though NCBRS is associated with various developmental and neurological abnormalities, ocular manifestations, particularly bilateral congenital glaucoma, are exceedingly rare, with only two other documented cases in the literature^[5,6]^. Diagnosis generally requires confirmation of a clinical suspicion by identifying a pathogenic variant in the *SMARCA2* gene through molecular genetic testing[7]. Here, we report another rare case of bilateral congenital glaucoma in a child diagnosed with NCBRS.

**HIGHLIGHTS**

- Rare case of bilateral glaucoma in a child with Nicolaides-Baraitser syndrome (NCBRS).
- First reported case of a Saudi Arabian patient with NCBRS and congenital glaucoma.
- Genetic testing confirmed the SMARCA2 mutation through whole-exome sequencing.
- Case highlights the need for early eye screening in NCBRS patients.

## Case description

**Figure 1.**
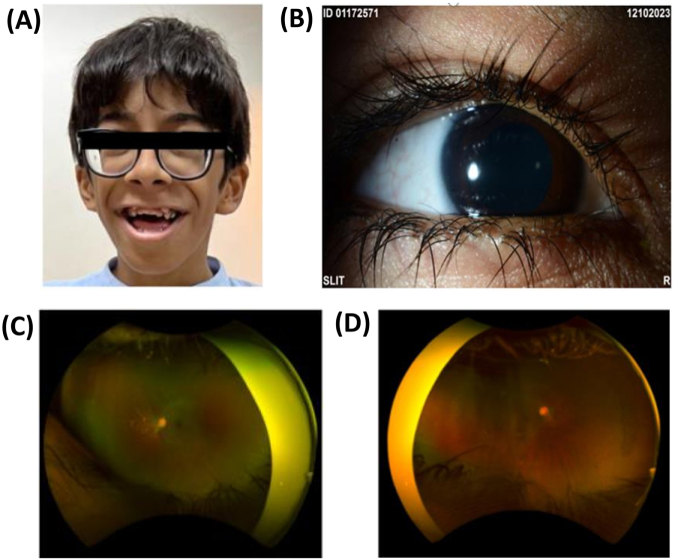
(**A**) External photo shows facial features consistent with NCBRS, including triangular shape, thick nares, broad philtrum, thin upper vermilion, thick everted lower vermilion, and wide mouth. (**B**) The slit-lamp photo shows hypertrichosis of the eyelashes with normal anterior segment structures. (**C**) Wide field color fundus photo of the left eye and (**D**) the right eye shows clear vitreous with a cup to disc ratio of 0.4 and no retinal pathology in both eyes. NCBRS, Nicolaides-Baraitser syndrome.

An 11-year-old male with high myopia and optic disc cupping in both eyes presented to the ophthalmology clinic. He had a history of primary congenital glaucoma, for which bilateral deep sclerectomy surgeries were performed during the neonatal period. The patient also exhibited attention deficit hyperactivity disorder and cognitive delay, with an intelligence quotient of 81. Physical examination revealed sparse hair on the scalp, and facial dysmorphic features included a triangular shape, thick nares, broad philtrum, thin upper vermilion, thick everted lower vermilion, and a wide mouth (as shown in Fig. 1A and 1B), short stature, prominent interphalangeal joints, kyphoscoliosis, generalized convulsive epilepsy, and hearing difficulties. Based on his clinical features, he was suspected to have NCBRS, a diagnosis later confirmed by genetic testing.

During the ophthalmic examination, best-corrected visual acuity with corrective lenses was measured at 20/60 in both eyes. Refraction results indicated severe myopia, with the right eye measuring −17.50−1.25 × 030 and the left eye −14.50−1.00 × 180. Intraocular pressure (IOP) was recorded as 20 mm Hg in the right eye and 16 mm Hg in the left eye, with central corneal pachymetry of 554 μm and 593 μm, respectively. A slit-lamp examination of the anterior segment was normal, with no iridectomies performed and flat superior blebs noted under the conjunctiva of both eyes. The dilated fundus examination showed a clear vitreous with a cup-to-disc ratio of 0.4, and no retinal pathology was detected in either eye, as shown in Figure 1C and 1D. Notably, the IOP remained controlled without the need for ocular medications.

Whole-exome sequencing identified a heterozygous likely pathogenic variant in the *SMARCA2* gene, specifically the c.3562 G>C (Ala1188Pro) mutation, confirming the diagnosis of autosomal dominant NCBRS.

## Discussion

This case of a child with bilateral congenital glaucoma provides further evidence of abnormal ocular features associated with the phenotypic spectrum of NCBRS. In addition, common features such as developmental delay, cognitive impairment, facial dysmorphism, musculoskeletal abnormalities, and hearing difficulty, previously documented in NCBRS cases, were all present in our patient^[1–3]^. Remarkably, NCBRS can be misdiagnosed as Williams syndrome in early childhood due to facial similarities. However, as patients age, the distinctive features of NCBRS become more pronounced[3]. Characteristic facial dysmorphisms include a triangular facial shape, dense eyelashes, a broad nasal base, thick nares, an upturned nasal tip, a rounded premaxilla, a broad philtrum, and a wide mouth. Additionally, palpebral fissures may be narrow and down slanting^[2,3]^. Hearing impairments, ranging from conductive hearing loss to congenital sensorineural deafness, have been reported in many patients[3].

The development of bilateral congenital glaucoma in our patient correlates strongly with a similar case reported by Sethi and colleagues[5]. Likewise, this group reported bilateral glaucoma and high myopia in a 12-year-old girl, along with cataract and degenerative vitreoretinopathy, which were absent in our case[5]. Similarly, congenital glaucoma was reported in an infant with hypertrophic cardiomyopathy in NCBRS[6]. These findings underscore the potential increased risk of ophthalmic complications among individuals with NCBRS. Besides, refractive errors of varying degrees, including myopia and astigmatism, are similar to previous reports and were also found in our presented case^[4,8]^. Our case expands the phenotypic spectrum of NCBRS, suggesting that congenital glaucoma might be underreported or not diagnosed in many of these patients. Therefore, more ophthalmological surveillance is needed to reveal the incidence of ocular abnormalities in NCBRS.

Identifying a heterozygous likely pathogenic variant in the *SMARCA2* gene, specifically the c.3562 G>C p.(Ala1188Pro) mutation in our case confirms the diagnosis of autosomal dominant NCBRS. Houdt and colleagues have previously reported this *de novo* mutation in NCBRS[7]. *SMARCA2* (previously known as *BRM*) is located on chromosome 9p24.3. It encodes the ATPase subunit of the SWI/SNF chromatin-remodeling complex. *SMARCA2* plays a crucial role in regulating gene expression by hydrolyzing ATP and altering chromatin accessibility. These processes are highly critical for cell differentiation, development, and the maintenance of various cellular functions[9].

Studies have previously shown that most mutations in NCBRS are missense or in-frame deletions, which cluster within the ATPase domain of the gene. These mutations do not impair the overall assembly of the SWI/SNF complex but disrupt the ATPase catalytic activity, leading to the clinical phenotypes observed in NCBRS^[7,10]^. ATP-dependent chromatin remodeling is one of the crucial controllers of the dynamic nature of chromatin. Considering the critical role of chromatin remodeling in gene expression and development^[11,12]^, we hypothesize that *SMARCA2* mutation(s) might disrupt these tightly regulated processes required for the proper development and function of ocular structures, e.g., impaired optic nerve development or retinal ganglion cell differentiation^[12,13]^, and could lead to congenital glaucoma or other ocular phenotypes. However, there is no direct evidence to link *SMARCA2* mutation(s) to these conditions and is needed further research.

To conclude, we believe that the ophthalmic manifestations observed in patients with NCBRS, such as bilateral glaucoma, may not be coincidental. Given the potential for serious ocular complications, clinicians should strongly consider recommending regular ophthalmologic evaluations for patients diagnosed with NCBRS. To our knowledge, this is only the third such report in the literature and the first case from the Arab population, specifically from Saudi Arabia.

## Data Availability

Not applicable.
